# The Problem for Hospital Staffs and the Colleges

**Published:** 1914-08-15

**Authors:** 


### HOSPITALS AND THE WAR.

### THE PROBLEM FOR HOSPITAL STAFFS AND THE COLLEGES.

The machinery for constituting the Territorial branch of the Royal Army Medical Corps is so closely bound up with the civilian system of medical practice that there is some danger lest the general public, and particularly the hospitals, will suffer from diversion of the energies and attentions which are customarily given to them. After all, the medical profession in this country is a comparatively small body, and at the present time there is reason to believe that room might well be found for a good many more qualified men than are available in the various branches of civilian practice, to say nothing of the military and special services; in regard to which the shortage of naval surgeons, referred to in The Hospital on more than one occasion recently, has for some little time represented an urgent need. Whilst men in general practice find it difficult to obtain assistants and *locum tenentes*, and whilst those who are willing to do such auxiliary work are able to command fees from 50 to 100 per cent. higher than obtained a few years ago, it cannot be doubted that there is room for a good many more young men as doctors in this country. Thus it has come to pass that at a time when the military services are making an unprecedented demand for qualified practitioners there are in proportion to general requirements fewer than has ever previously been the case. Military service with its adventures and opportunities is a strong attraction to junior men, and just now there is a real danger that the calls of patriotism will result in a shortage of doctors for the general needs of the public, a circumstance that may quite quickly have very serious consequences. Already one hears of a leading general hospital where the keenness of the residents to volunteer as civil surgeons has been such that the governing committee have been compelled to exercise their veto, and permit only a few to go, these favoured ones being chosen by ballot; the proper services of the institution being thus threatened before any question had arisen of the staff being depleted to meet the requirements of the Medical Corps attached to the Territorial Army.

Until the Territorial base hospitals allotted to the London area are established it does not seem likely that the metropolitan hospitals will be embarrassed by loss of actual staff surgeons, and at the time of writing they have in this respect felt chiefly the calling out of those men who are attached to the Special Reserve of the R.A.M.C. and other officers who have to mobilise simultaneously with the Regulars. Harley Street and neighbourhood had many thrills last week when various well-known practitioners received their sudden calls to active service, calls which, be it noted, left little or no time for handing over either hospital or private work to the care of friends or colleagues. But, nevertheless, the places of these men must be filled if the public are not to suffer, and in view of the far more serious depletion of staffs that must inevitably occur if several Territorial London hospitals are established, it certainly behoves committees of management to make some provision for maintaining the efficiency of the important institutions under their control.

Judging from the wave of patriotic enthusiasm and keenness to serve the Army in one capacity or another that is sweeping over the hospitals, such provision may not be secured without difficulty unless immediate steps be taken. Not only are many members of hospital staffs liable to be called out to join the Territorial General Hospitals, but there is evidence of not a few semi-private plans to constitute hospitals and ambulance corps under the control of hospital physicians and surgeons, although it is difficult to believe that, under the circumstances likely to prevail, the authorities will see their way to release men other than those officially called upon at the outset. The air seems full of schemes for ambulance units, including hospital accommodation, for various suburban and provincial centres of hospital relief for the wounded, for hospital ships, and so forth. But it cannot be supposed for a moment by those who are most competent to deal with these matters that the London voluntary hospitals can possibly provide staffs for all these potential war organisations, and at the same time adequately provide for the needs of our vast Metropolitan population. The resulting position would appear to depend largely on the number of qualified men who are secured as civil surgeons by the Army and Navy; for, although it is clear that hospital authorities can stop wholesale desertion by their residents, they certainly have no control over the numerous young freshly-qualified diplomates who, in the ordinary way, are just now to be found doing locum work, or waiting for vacancies amongst the house physicians, house surgeons, resident obstetric officers, or house anæsthetists. A brief and somewhat restricted inquiry in another direction quickly afforded evidence that amongst those holding the useful appointments of clinical assistants there is a similar tendency to desert civilian work for the time being; but one is glad to know definitely that at one leading hospital at least the governing body has made it known that responsible duties cannot thus be left at a moment's notice, and that posts cannot be kept open for those clinical assistants who feel it incumbent upon them to seek military occupation at the present juncture. However, it cannot be doubted that the prospect has to be faced of many resident hospital officers wishing to offer their services to the Admiralty or War Office the moment their term of office comes to an end. And thus it is that, both in respect of staff work and of routine, subordinate duties in wards and out-patients' departments—of a kind, be it noted, that is absolutely vital to the efficient working of the hospitals—these institutions may be seriously embarrassed if either the Territorial hospitals call for all those surgeons who are attached to them, or if the Military Services should require a very large number of civilian medical officers. Should the war be so prolonged, or its results so grim that in both these directions very large demands are made upon the medical profession, it is difficult to see how the hospitals will be able to carry on in their accustomed manner. One thing is quite certain, and that is, the hospitals will inevitably be confronted with more, rather than less, work under existing and probable circumstances. It will be a most surprising thing if the stress and strain of war on the civilian population does not considerably increase the amount of illness above the average in the next six months. Even on the most moderate estimates it would appear that the hospitals, if they are to maintain their stereotyped high standard of efficiency throughout the trials that are before us, must inevitably have to make up loss of skilled officers due to the following sources:—

1. Service of members of staffs with (*a*) Special Reserve of R.A.M.C., and (*b*) Territorial Hospitals.
2. Volunteering of newly qualified men, residents finishing terms of office, and clinical assistants as civilian surgeons.
3. Service and volunteering of nurses—a matter which, equally with the doctors' problem, requires the most careful consideration of hospital authorities.
4. The calling out of administrative officials who are attached to the Territorial Force as combatants or in other capacities.

### Attitude of Students.

Let us see now from what sources the hospitals can possibly reinforce the ranks of qualified men upon whom they have to rely in their campaign against sickness. Clearly they have only the students of the medical schools to fall back upon, and inquiry as to what attitude these will take shows that it is by no means a matter of course that the medical students will consistently take the place of their seniors. For one thing, there is no doubt that the numbers of students available for more responsible work than reading for examinations will be largely reduced by volunteering for work as dressers and assistants to the various ambulance corps. Certainly many of those whose chance of qualifying in the immediate future is remote will do their best to join the forces as first-aid officers and assistants to the surgeons. The opportunities thus afforded of seeing something of life and its wider aspects are too exceptional to be neglected, and, after all, it says much for the spirit of our race that its youthful members are as desirous, as they undoubtedly are, of taking some active part in the military measures now going forward. It would be interesting to know, for example, how many medical students who happen to be the fortunate possessors of motorcycles are at the present moment trying their hardest to obtain work in which they can make use of their iron steeds for Government purposes.

There is another obvious source of depletion in regard to the services of medical students likely to be available for the hospitals during the present struggle; this is, of course, the offer of commissions in combatant corps to well-educated young men of the university or technical school class. Notices are already up in the medical schools pointing out the eligibility of many students for these commissions, and it cannot be doubted that numbers will embrace this opportunity of covering themselves with military glory.

### Expediting Examination Facilities.

That the dearth of hospital officers may become serious is evidently becoming realised in responsible quarters, and it is interesting to note that the suggestion has been made by the Vice-Chancellor of London University that not only should the process of qualification be expedited for senior students just now, but that junior men should be taken on by the hospitals as ward clerks and dressers at a very much earlier time in their careers than is usual. He advocates, indeed, something in the nature of a return to the old apprentice system, in which the student had the advantage of clinical work from the outset of his studies. Possibly some temporary arrangement of this kind might relieve the situation, but the fact must not be overlooked that this same expediting of examination facilities is also being looked to in other quarters for providing a sufficiency of civilian surgeons should emergency arise. In any case, the speeding up process could scarcely be made conditional on candidates, either refraining from or volunteering for the post of civilian surgeon. It must not be supposed that the hospitals must inevitably find themselves in a hopeless position shortly owing to the dearth of doctors and students. But careful arrangements made now will almost certainly tide over any such difficulty that is likely to arise in the course of the next few months. Forewarned is forearmed; hence a review of possibilities and present tendencies is absolutely necessary now, so that a considered course of action may be taken to avoid an *impasse* by every corporation and college that is concerned with medical education and the staffing of the metropolitan hospitals at the present time.

### The Depletion of Medical Staffs.

A moment's thought shows that the hospitals have already vastly assisted the country in its preparations for war, in that every doctor, every nurse, every ambulance officer, and every dresser, directly or indirectly, owes his or her skill to these institutions. And now that they are threatened by serious depletion in regard to *personnel*, it is evident that their needs in the immediate future are likely to be greater than ever. Certainly the hospitals will not require less, but more, monetary assistance from the charitable public than usual. Let the public realise that the voluntary hospitals are a firm rock upon which rests the whole fabric, not only of civilian practice and nursing, but of the surgical and sanitary work of the military services. In this way the position may become properly understood, and the foolish though dangerous schemes of well-meaning but amateur philanthropists will then be rendered harmless at the outset. The hospitals will need all the support they can get.

